# Determination of the Quality of Oil Obtained from Protein Hydrolysate Produced Using Rainbow Trout (*Oncorhynchus mykiss*) By-Products

**DOI:** 10.3390/foods14183227

**Published:** 2025-09-17

**Authors:** Koray Korkmaz, Serpil Öztürk

**Affiliations:** Fatsa Faculty of Marine Sciences, Department of Fisheries Technologies, Ordu University, 52400 Ordu, Turkey; serpilozturk52@gmail.com

**Keywords:** lipid quality, fish protein hydrolyzate, by-product, circular bioeconomy, waste fish oil, rainbow trout

## Abstract

The growing demand for sustainable food sources requires the efficient use of aquaculture by-products. This study aimed to optimize enzymatic hydrolysis conditions for the simultaneous recovery of fish protein hydrolysate (FPH) and oil from rainbow trout (*Oncorhynchus mykiss*) processing by-products. Hydrolysis was performed at different temperatures (30–50 °C), enzyme concentrations (0.5–1.5%), and durations (30–90 min), and the optimal conditions were determined as 40 °C, 1% enzyme concentration, and 60 min. Under these conditions, oil yield reached 11.46%, while quality indices remained within acceptable limits (peroxide value: 1.78–3.47 meq O_2_/kg; thiobarbituric acid reactive substances: 0.41–1.41 mg MDA/kg; free fatty acids: 0.27–4.12%). Fatty acid analysis revealed 22.5% saturated, 46.31% monounsaturated, and 23.52% polyunsaturated fatty acids, including notable levels of EPA and DHA. The protein hydrolysates obtained under optimized conditions contained 22.61% protein and essential amino acids, accounting for 52.4% of the total amino acid content, confirming their high nutritional value. Overall, the findings demonstrate that rainbow trout by-products can be effectively valorized through enzymatic hydrolysis to produce oil and protein hydrolysates of acceptable quality, which may serve as alternative ingredients for food and feed applications.

## 1. Introduction

Ensuring sufficient food production for the projected global population of 9.6 billion by 2050 is one of the greatest challenges facing humanity [1]. Fisheries and aquaculture play a crucial role in global food security, providing around 17% of the world’s protein supply and representing one of the fastest-growing food sectors [2,3]. However, the expansion of production also generates significant amounts of processing by-products, estimated at 27.85 million tons annually [4]. These by-products, including heads, viscera, bones, and skin, are often discarded or underutilized, despite their potential as sources of valuable compounds [5,6]. Within the framework of the circular and blue economy, efficient valorization of fish by-products is essential to reduce waste and create new value chains [7,8]. Recent studies have shown that fish waste can be converted into high-value products such as protein hydrolysates, polyunsaturated fatty acids (PUFA), bioactive peptides, and fish oil, all of which have applications in food, feed, pharmaceuticals, fertilizers, and even biofuels [9,10,11]. The global market for fish oil alone is projected to reach USD 2.8 billion by 2027, largely driven by aquaculture demand [12,13].

Among these resources, fish viscera represent a promising raw material, containing both proteins and oils rich in PUFA. Enzymatic hydrolysis is an efficient method to recover protein hydrolysates while simultaneously releasing oil fractions of high nutritional and functional value [14,15]. Valorization of such by-products, therefore, contributes not only to sustainable waste management but also to human health and economic growth.

In this study, rainbow trout by-products were hydrolyzed enzymatically under different process conditions to optimize protein hydrolysate production. The oil obtained as a co-product was also analyzed to evaluate its potential as a functional food ingredient. The aim was to develop a model for sustainable utilization of fish processing by-products, supporting both the circular bioeconomy and the production of high-value compounds for human and animal nutrition.

## 2. Materials and Methods

### 2.1. Materials

Rainbow trout (*Oncohoryncus mykiss*) processing wastes, which are the most common aquaculture by-products in Turkey, were collected from local aquaculture enterprises in Ordu province. Samples were transported under cold chain conditions to the Processing Technology Laboratory, Department of Fisheries Technology Engineering, Ordu University Fatsa Faculty of Marine Sciences, and stored at −40 °C (Premium U410, New Brunswick Scientific, Eppendorf AG, Co., Inc., Framingham, MA, USA) until analysis.

### 2.2. Enzymatic Hydrolysis

Enzymatic hydrolysis of trout by-products and optimization values were performed according to [16]. Frozen by-products (stored in 5 kg packages) were thawed at room temperature, minced using a meat grinder (Empero, EMP.12.01.P, Konya, Turkey), and heated in a water bath (Memmert WNB 22, Schwabach, Germany) at 90 °C for 20 min to inactivate endogenous enzymes. The material was cooled and homogenized (Ultra-Turrax T25, IKA, Staufen, Germany) with distilled water at a 1:1 ratio (*w*/*v*).

Hydrolysis was carried out using a commercial protease (Flavourzyme^®^, Novozymes A/S, Bagsvaerd, Denmark). Hydrolysis conditions were varied at three enzyme-to-substrate ratios (0.5%, 1.0%, and 1.5% *w*/*w*), three temperatures (30, 40, and 50 °C), and three hydrolysis times (30, 60, and 90 min). The reaction pH was maintained at 7.0 using 2 N NaOH (Mettler Toledo, Greifensee, Switzerland). Hydrolysis was terminated by heating the mixture to 90 °C for 5 min, followed by 15 min cooling.

After hydrolysis, samples were centrifuged at 4100 rpm for 20 min (Sigma 3–30 KS, Darmstadt, Germany). Four phases were obtained: (i) oil phase, (ii) light protein–oil phase, (iii) aqueous protein phase, and (iv) insoluble residue. The oil phase was carefully separated and collected for further analyses.

### 2.3. Proximate Composition

Moisture content was determined by oven drying at 105 °C to constant weight [17]. Crude protein was analyzed by the Kjeldahl method AOAC 1998 [18], using a nitrogen-to-protein conversion factor of 6.25. Crude fat was quantified using the Bligh and Dyer [19] chloroform–methanol extraction method (2:1 *v*/*v* solvent ratio). Ash content was determined by incineration at 550 °C in a muffle furnace [20].

### 2.4. Determination of Oil Yield

Oil yield was calculated gravimetrically according to Samaranayaka and Li-Chan [21]:

Oil Yield (%) = (Weight of oil (g)/Weight of hydrolysate (g)) × 100

### 2.5. Quality Analyses of Hydrolysate Oil

#### 2.5.1. Determination of Peroxide Value (PV)

PV was determined using the iodometric titration method [22]. Results were expressed as milliequivalents (meq) of active oxygen per kg oil.

#### 2.5.2. Determination of Thiobarbituric Acid (TBA) Value

Secondary lipid oxidation products (malondialdehyde equivalents) were measured spectrophotometrically at 532 nm following the Tarladgis et al. [23] procedure.

#### 2.5.3. Determination of Free Fatty Acids (FFA)

FFA content was quantified by titration with 0.1 N NaOH using phenolphthalein indicator and expressed as % oleic acid [24].

#### 2.5.4. Fatty Acid Profile

Fatty acid methyl esters (FAMEs) were prepared by transesterification with 2% H_2_SO_4_ in methanol and analyzed by gas chromatography (GC-FID, Agilent Technologies, Santa Clara, CA, USA) equipped with a capillary column (DB-23, 60 m × 0.25 mm × 0.25 µm). Injector and detector temperatures were set at 250 °C, oven temperature program started at 140 °C (held 5 min), increased to 230 °C at 4 °C/min, and held for 10 min. Helium was used as the carrier gas at a flow rate of 1 mL/min. Fatty acids were identified by comparing retention times with authentic standards (Supelco 37 Component FAME Mix, Sigma-Aldrich, St. Louis, MO, USA).

### 2.6. Statistical Analysis

All experiments were performed in triplicate. Results were expressed as mean ± standard deviation. Statistical analyses were conducted using SPSS version 25.0 (IBM Corp., Armonk, NY, USA). Differences among groups were evaluated by one-way analysis of variance (ANOVA), followed by Tukey’s post hoc test. A significance level of *p* < 0.05 was considered statistically significant.

## 3. Results and Discussions

### 3.1. Rainbow Trout (Oncorhynchus mykiss) and Nutrient Composition of Waste

The proximate composition of rainbow trout (*Oncorhynchus mykiss*) fillet and processing by-products (head, trimmings, viscera, and skeleton) is presented in Table 1. In the fillet samples, lipid, protein, ash, and moisture contents were 1.39%, 17.78%, 1.14%, and 77.74%, respectively, whereas the corresponding values for the by-products were 23.10%, 14.10%, 3.70%, and 59.10%. These findings indicate that trout waste contains considerably higher lipid and ash fractions compared to edible muscle tissue, highlighting its potential as a raw material for oil recovery and mineral extraction.

The nutritional composition of fish by-products is strongly influenced by species and tissue type. For instance, Suvanich et al. [25] reported that mackerel contained the highest lipid level (11.7%) among the species examined, whereas cod exhibited the lowest value (0.1%). Similarly, salmon muscle showed the greatest protein concentration (23.5%), while sole had the lowest (14%). Moisture contents generally ranged from 69% to 84.6%, which is consistent with the present data. Other studies have also documented considerable variation depending on anatomical origin: salmon dark muscle contains 12.5% lipid and 17.5% protein, while its white muscle is richer in protein (20.4%) but poorer in fat [26].

Our results also confirm previous reports on trout and other commercial species. Korkmaz [10] found trout wastes to be particularly rich in lipids (22.11%), a value that surpasses those of haddock (6.06%) and anchovy (7.23%). Anchovy wastes, however, were richer in ash (4.0%), consistent with Koç [27], who reported values of 73.85% moisture, 14.54% crude protein, 6.60% fat, and 5.00% ash for anchovy by-products. Comparable observations were made by Roslan et al. [28] for tilapia wastes and by Esteban et al. [29] in their survey of fish-selling enterprises, which revealed by-products to be important reservoirs of protein and minerals.

Beyond macronutrients, fish by-products provide valuable compounds such as polyunsaturated fatty acids, amino acids, bioactive peptides, and minerals. For example, Korkmaz and Tokur [30] demonstrated that Israeli carp viscera contained the highest lipid content (22.38%) among the examined tissues, whereas scales were richest in protein (21.31%). Such variability illustrates the importance of anatomical source in determining the value of fish waste. Moreover, viscera and skeletal tissues are often enriched in monounsaturated fatty acids such as oleic and palmitic acids [31,32].

Overall, the present findings support the view that rainbow trout by-products are a nutrient-dense resource, with lipid levels exceeding those of the edible portion. This composition makes them attractive substrates for the production of value-added products, particularly fish oil and protein hydrolysates, within a circular bioeconomy framework.

### 3.2. Oil Yield

Fish oil can be extracted by several methods, including low-temperature, high-temperature, and enzymatic approaches. Among these, enzymatic hydrolysis has been widely recognized as the most effective due to its ability to release lipids at relatively mild conditions, thereby limiting oxidative damage [33]. In the present study, rainbow trout by-products were hydrolyzed at three temperatures (30, 40, and 50 °C), with enzyme concentrations of 0.5%, 1.0%, and 1.5% (APU/g), and reaction times of 30, 60, and 90 min. Under these conditions, oil yield ranged from 5.75% to 11.46% Table 2.

Statistical analysis indicated that all three factors—temperature, enzyme concentration, and hydrolysis time—had a significant influence on oil recovery (*p* < 0.05). The optimal yield was obtained at 40 °C, 1% enzyme concentration, and 60 min of hydrolysis. These conditions were, therefore, selected for subsequent fatty acid profiling. At the temperature level, oil yields at 40 °C were significantly higher than those at 30 °C, whereas 50 °C produced intermediate values. Increasing enzyme concentration also enhanced oil recovery, with yields at 1% and 1.5% being statistically similar but significantly higher than those at 0.5%. Likewise, prolonging hydrolysis time from 30 to 60 or 90 min significantly improved lipid release.

These findings are in line with previous reports on fish waste utilization. For instance, Purnamayati et al. [34] reported oil yields of 15.6–33.9% from tilapia viscera, with extraction efficiency strongly influenced by temperature and reaction duration. Similarly, Suseno et al. [35] and Nazir et al. [36] demonstrated that both time and temperature were critical determinants of oil recovery from tilapia by wet processing, achieving maximum yields of 6.44% and 12.8%, respectively. Taken together, these studies confirm that the extraction environment plays a pivotal role in determining lipid yield.

From a mechanistic standpoint, higher temperatures promote the mechanical separation of solid and liquid fractions, disrupt adipose tissues, and accelerate the diffusion of lipids into the aqueous phase. Enzyme-assisted hydrolysis enhances this process by breaking down protein matrices that entrap fat droplets, thereby improving oil liberation even at lower temperatures [37,38]. Enzymatic methods, therefore, offer advantages over conventional high-temperature extraction, which may cause thermal degradation, and over wet processing, which typically yields lower-quality oils.

Overall, our results confirm that enzymatic hydrolysis not only improves lipid yield but also provides a gentler processing environment that preserves oil quality. This makes enzymatic extraction a promising technology for valorizing fish processing by-products in line with circular bioeconomy principles.

### 3.3. Quality Analysis of Hydrolysate Oil

During enzymatic hydrolysis of rainbow trout by-products, the lipid phase obtained as a co-product was assessed in terms of oxidative stability and quality indicators, including peroxide value (PV), thiobarbituric acid reactive substances (TBARs), and free fatty acids (FFA). These parameters provide critical information on the freshness, stability, and nutritional integrity of the recovered oil.

#### 3.3.1. Peroxide Value (PV)

PV is a primary measure of lipid oxidation and reflects the accumulation of peroxides during processing. In this study, PVs varied significantly with hydrolysis time, increasing as the duration extended from 30 to 90 min (*p* < 0.05) (Table 3). By contrast, enzyme concentration and processing temperature did not exert a statistically significant effect (*p* > 0.05). At 30 °C, PV ranged between 2.15 and 3.12 meq O_2_/kg, while at 40 and 50 °C the values extended to 3.46 and 3.47 meq O_2_/kg, respectively. These findings suggest that prolonged exposure during hydrolysis accelerates lipid oxidation, independent of temperature.

Our results are consistent with reports by Özyurt et al. [39], who observed PVs of 2.12 meq O_2_/kg in oils derived from sea bass by-products, and with Monsiváis-Alonso et al. [40], who reported values near 8.65 meq O_2_/kg in tuna oil. Importantly, all PVs in this study remained below the Codex Alimentarius and EFSA thresholds of 10 meq O_2_/kg for edible oils [41,42]. Thus, despite some oxidative increase with time, the oils remained within acceptable quality standards.

#### 3.3.2. Tiyobarbitürik Acid Reactive Substances (TBARs)

TBARs values represent secondary oxidation products, primarily malondialdehyde, which are indicative of rancidity. In the present study, TBARs ranged from 0.41 to 1.41 mg MA/kg, with significant differences detected across both enzyme ratios and hydrolysis times (*p* < 0.05) (Table 4). At 30 °C, differences were minimal; however, at 40 °C and 50 °C, the 1.5% enzyme treatment at 90 min exhibited significantly higher TBARs values, indicating that both temperature and enzyme activity can accelerate secondary oxidation when combined with extended hydrolysis.

These observations align with previous research. For example, Kaitaranta [43] suggested that oils exceeding 19 mmol MDA/1000 g should be considered unacceptable, whereas Schormüller [44] proposed a maximum threshold of 3 mg MA/kg for high-quality material. The TBARs values in this study fell well below these critical levels, confirming the suitability of the oil for potential use in food or feed applications.

#### 3.3.3. Free Fatty Acids (FFA)

FFA levels serve as an indicator of hydrolytic rancidity and enzymatic lipolysis. In our results, FFA ranged between 0.25% and 4.12% (as oleic acid), with significant differences observed at certain enzyme ratios and hydrolysis times (*p* < 0.05) (Table 5). The highest value (4.12%) was detected at 30 °C with 1.5% enzyme for 30 min, while other treatments generally fell within the recommended range of 1–7% set by the International Fishmeal and Oil Manufacturers Association [37]. Importantly, most treatments produced FFA levels below the stricter 3% threshold recommended for edible oils [45].

These findings mirror earlier observations that viscera-derived oils often contain higher FFA due to the presence of endogenous lipases and the susceptibility of PUFA-rich lipids to hydrolysis [46]. Nonetheless, values in the present study remained within internationally accepted limits, demonstrating that enzymatic hydrolysis can yield oils of acceptable quality even when by-products are used as raw materials.

#### 3.3.4. Fatty Acids

According to the optimization results of the fish protein hydrolysate oil obtained as a result of the study, 40 °C, 1% enzyme ratio, and 1 h hydrolysis time were determined as optimum results and evaluated in terms of fatty acid profile.

**Figure 1:**
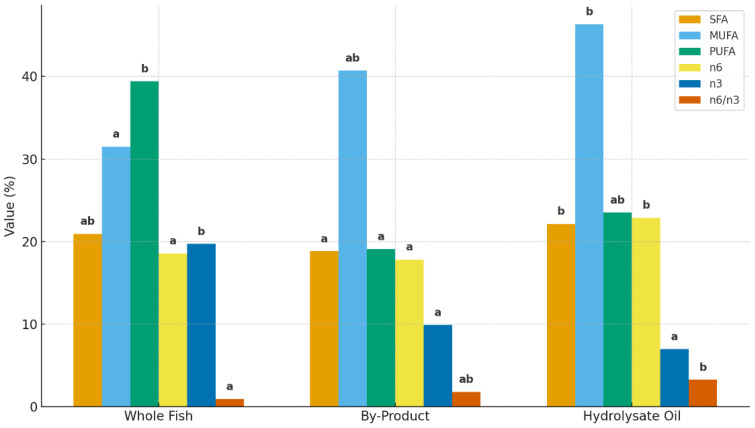
Fatty acid profile of whole fish, by-product, and optimized hydrolysate oil.Different letters (a, b) indicate significant differences (*p* < 0.05).

The fatty acid composition of the oil from trout meat, waste, and protein hydrolysate produced by optimization with the enzymatic hydrolysis method is shown in Table 6 and Figure 1.

According to the results obtained in this study, SFA ratios were 20.94% in oils obtained from fillet fish, 18.81% in oils obtained from fish by-products, and 22.15% in fish protein hydrolysate oil obtained as a result of optimization. Similarly, Gamsız et al. [47] found SFA ratios between 15.57 and 33.38% in oils obtained from whole-bodied fish and 16.3–31.89% in oils obtained from fish waste. In addition, the MUFA ratio was 31.48% in oils obtained from fillet fish, 40.72% in fish by-product oils, and 46.31% in fish protein hydrolysate oil obtained as a result of optimization. Similarly, Gamsız et al. [47] found MUFA ratios between 24 and 38.69% in oils obtained from whole-bodied fish and between 25.81 and 47.57% in waste oils. PUFA ratio was found to be 39.39% in oils obtained from filleted fish, 19.11% in trout hydrolysate oil, and 23.52% in trout hydrolysate oil obtained as a result of optimization.

In fish feeding, fats are not only a source of energy but also play a role in the delivery of essential fatty acids to the fish. Especially, high-chain unsaturated fatty acids are needed in the diet of marine fish. When we look at the ratios of DHA and EPA, which are among the most important PUFAs in terms of nutrition, the DHA ratio was 18.57% in the oils obtained from all fish, 17.83% in the oil obtained from fish by-products, and 22.89% in the fish protein hydrolysate oil obtained as a result of optimization. EPA ratios were found to be 19.71% in oils obtained from whole fish, 9.92% in fish by-product oils, and 6.99% in trout hydrolysate oil obtained as a result of optimization. In addition, the n6/n3 ratio was found to be 0.95 in oils obtained from whole-bodied fish, 1.80 in fish by-product oils, and 3.27 in fish protein hydrolysate oil obtained as a result of optimization.

Özyurt et al. [39] investigated the fatty acid composition and oxidative stability of oils obtained from sea bass by-products as well as acid silage and bacterial fermentation of fish and found PUFA contents between (23.27–23.64%). Monsiváis-Alonso et al. [40] found the fatty acid results of tuna oil as MUFA 13.90% and PUFA 38.80%. The results revealed the presence of significant amounts (26.90%) of ω-3 PUFA, especially docosahexaenoic acid (C20:5 ω-3, DHA), indicating that it is possible to recover and utilize this oil.

Giogios et al. [48] examined the fatty acid composition of different fish oils and found that SFA values ranged between 19.1% and 28.3%, MUFA values ranged between 26.3% and 48.3%, and PUFA values ranged between 28.7% and 44%.

Durmuş [49] determined that the fatty acid compositions of edible meats of 13 different seafood species caught in the Northeastern Mediterranean coast ranged from 27.68% to 36.59% saturated fatty acids, 8.99% to 35.84% monounsaturated fatty acids, and 10.69% to 39.57% polyunsaturated fatty acids.

According to the results of the study, the DHA content of oils produced from whole fish was higher than that of oils obtained from processing waste. The main reason for this is that these oils are obtained from fish species called pelagic and oily fish. Alkio et al. [50] reported that 18.3% of tuna oil was DHA. The DHA content of 22.89% in our study was similar to that of Alkio et al. [50]. Many researchers have reported the DHA content of tuna oil at different rates. However, these values generally vary between 9.3% and 25%. These differences are reported to be due to differences in the feeding environment of tuna [48,51,52,53]. Gamsız et al. [47] found that EPA ratios ranged between 8 and 9.89% in oils obtained from whole fish and between 2.63 and 15.28% in fish by-product oils. In our study, EPA ratios similar to the literature were determined as 6.99% in trout hydrolysate oil obtained as a result of optimization. Selmi et al. [54] examined the fat and fatty acid compositions of waste viscera of tuna caught at different times of the year and reported that fat content ranged between 1.49% and 14.26% and EPA values ranged between 4% and 14.26%.

Highly unsaturated fatty acids, especially DHA, EPA, and ArA, are involved in the structure and maintenance of cell membranes and act as precursors for the secretion of steroid hormones and prostaglandins. These fatty acids are, therefore, essential for the survival of fish as well as other living organisms. The ability of freshwater fish to convert the highly unsaturated fatty acids linolenic acid into DHA and EPA and linoleic acid into ArA suggests that it is possible to use terrestrial fats in the diet of freshwater fish. However, sources with high HUFA content should be added to fish feeds to meet the needs of saltwater fish [55,56].

In general, reducing the use of fish oil in fish feed is essential for sustainable aquaculture. However, valuable new sources need to be found to meet the high unsaturated fatty acid requirements of fish. The first sources that come to mind are aquatic organisms. In particular, phytoplankton and zooplankton species (copepods), krill species, and protozoa are sources of highly unsaturated fatty acids thought to be derived from aquatic areas. In addition, transgenic studies have attempted to transfer genes involved in the synthesis of long-chain fatty acids in marine algae to terrestrial oilseed crops to provide DHA and EPA [57,58,59,60,61]. Linder et al. [62] reported that some phytoplankton species contain 14.97% DHA and 9.28% EPA, while krill species contain 15.8–28.3% DHA and 6.7–31.6% EPA. Yeşilsu and Özyurt [63] reported that the EPA ratio of anchovy oil was 10.21% and the DHA ratio was 18.10%. Goosen et al. [64] reported that rainbow trout silage oil is a cost-effective alternative dietary oil for tilapia diets with advantages over some traditional fish oils.

Researchers Mgbechidinma et al. [65], reported that the average nutritional quality values of fatty acids of waste crude fish oil were as follows: Nutritive value index (0.87–1.68), health beneficial index (2.19–2.82), fish lipid quality (24.18–28.88), polyunsaturated/saturated (0.85–1.33), omega-6/omega-3 (0.18–0.22), atherogenic index (0.35–0.46), thrombogenic index (0.23–0.33), hypocholesterolemic/hypercholesterolemic ratio (2.21–2.77).

## 4. Conclusions

This study demonstrated that rainbow trout (*Oncorhynchus mykiss*) by-products can be effectively valorized through enzymatic hydrolysis to produce both fish oil and protein hydrolysates. Optimization identified 40 °C, 1% enzyme concentration, and 60 min as the most suitable conditions, resulting in high oil yield and acceptable quality parameters.

The findings confirm that trout processing waste is a valuable raw material for alternative lipid sources and functional products, supporting sustainable aquaculture and circular bioeconomy goals. Overall, enzymatic hydrolysis offers an efficient strategy to reduce waste, improve resource utilization, and contribute to the development of value-added products from fish by-products.

## Figures and Tables

**Table 1:** Nutrient composition of rainbow trout meat and waste.

Nutrient Elements (%)	Rainbow Trout Meat	Rainbow Trout Waste
**Lipid**	1.39 ± 0.17 ^a^	23.10 ± 0.72 ^b^
**Protein**	17.78 ± 0.33 ^a^	14.10 ± 0.45 ^a^
**Ash**	1.14 ± 0.34 ^a^	3.70 ± 0.19 ^a^
**Moisture**	77.74 ± 0.44 ^b^	59.10 ± 0.87 ^a^

± Standard deviation is shown. *n* = 3. Different letters (^a,b^) in the same column indicate significant differences (*p* < 0.05).

**Table 2:** Fish protein hydrolyzate oil yield (%).

Temperature (°C)	Time (min)	Enzyme Ratio (%)		
		0.5	1	1.5
**30**°	30′	6.80 ± 0.02 ^b1^	5.83 ± 0.02 ^b2^	7.18 ± 0.04 ^b2^
	60′	7.10 ± 0.04 ^a1^	5.75 ± 0.02 ^a2^	7.17 ± 0.05 ^a2^
	90′	6.65 ± 0.04 ^a1^	6.42 ± 0.05 ^a2^	8.75 ± 0.15 ^a2^
**40**°	30′	9.16 ± 0.15 ^b1^	11.46 ± 0.40 ^b2^	10.94 ± 0.05 ^b2^
	60′	9.10 ± 0.10 ^a1^	10.20 ± 0.23 ^a2^	11.02 ± 0.23 ^a2^
	90′	9.68 ± 0.19 ^a1^	11.12 ± 0.31 ^a2^	11.20 ± 0.30 ^a2^
**50**°	30′	9.55 ± 0.06 ^b1^	8.64 ± 0.16 ^b2^	9.60 ± 0.21 ^b2^
	60′	9.52 ± 0.13 ^a1^	8.93 ± 0.21 ^a2^	9.29 ± 0.26 ^a2^
	90′	8.12 ± 0.22 ^a1^	9.60 ± 0.03 ^a2^	9.77 ± 0.09 ^a2^

± Standard deviation is shown. *n* = 3. Different letters (^a,b^) in the same column indicate significant differences between times (*p* < 0.05). Different numbers (^1,2^) in the same row indicate significant differences between enzyme ratios (*p* < 0.05).

**Table 3:** PV (mEq/kg) values of hydrolyzate fish oil.

Temperature (°C)	Time (min)	Enzyme Ratio (%)		
		0.5	1	1.5
**30**°	30′	2.15 ± 0.03 ^a1^	2.15 ± 0.27 ^a1^	2.26 ± 043 ^a1^
	60′	2.26 ± 0.14 ^a1^	2.37 ± 0.29 ^a12^	2.68 ± 0.14 ^a1^
	90′	2.92 ± 0.27 ^a2^	3.04 ± 0.17 ^a2^	3.12 ± 0.30 ^a1^
**40**°	30′	1.87 ± 0.12 ^a1^	1.96 ± 0.29 ^a1^	1.89 ± 0.42 ^a1^
	60′	2.46 ± 0.15 ^a2^	2.66 ± 0.15 ^a2^	2.48 ± 0.13 ^a12^
	90′	3.41 ± 0.12 ^a3^	3.46 ± 0.10 ^a3^	3.22 ± 0.19 ^a2^
**50**°	30′	2.25 ± 0.23 ^a1^	1.97 ± 0.30 ^a1^	1.78 ± 0.00 ^a1^
	60′	2.27 ± 0.18 ^a1^	2.36 ± 0.28 ^a1^	2.46 ± 0.14 ^a2^
	90′	3.23 ± 0.08 ^a2^	3.47 ± 0.13 ^a2^	3.43 ± 0.11 ^a3^

± Standard deviation is shown. *n* = 3. Different letters (^a,b^) in the same column indicate significant differences between times (*p* < 0.05). Different numbers (^1,2,3^) in the same row indicate significant differences between enzyme ratios (*p* < 0.05).

**Table 4:** TBARs values of hydrolysate fish oil (mg MA/kg).

Temperature (°C)	Time (min)	Enzyme Ratio (%)		
		0.5	1	1.5
**30**°	30′	0.59 ± 0.04 ^a2^	0.58 ± 0.08 ^a1^	0.51 ± 0.07 ^a2^
	60′	0.49 ± 0.04 ^a12^	0.52 ± 0.04 ^a1^	0.55 ± 0.08 ^a2^
	90′	0.48 ± 0.10 ^ab1^	0.55 ± 0.09 ^b1^	0.41 ± 0.04 ^a1^
**40**°	30′	0.74 ± 0.08 ^a1^	0.55 ± 0.02 ^a1^	0.75 ± 0.22 ^a1^
	60′	1.04 ± 0.30 ^a1^	0.70 ± 0.08 ^a2^	0.87 ± 0.58 ^a12^
	90′	0.95 ± 0.46 ^a1^	0.72 ± 0.14 ^a2^	1.41 ± 0.09 ^b2^
**50**°	30′	1.28 ± 0.23 ^a1^	1.24 ± 0.51 ^a1^	0.97 ± 0.09 ^a2^
	60′	0.84 ± 0.11 ^a1^	1.05 ± 0.49 ^a1^	0.85 ± 0.13 ^a12^
	90′	1.12 ± 0.58 ^a1^	0.73 ± 0.03 ^a1^	0.71 ± 0.08 ^a1^

± Standard deviation is shown. *n* = 3. Different letters (^a,b^) in the same column indicate significant differences between times (*p* < 0.05). Different numbers (^1,2^) in the same row indicate significant differences between enzyme ratios (*p* < 0.05).

**Table 5:** FFA values of hydrolysate fish oil (% oleic acid).

Temperature (°C)	Time (min)	Enzyme Ratio (%)		
		0.5	1	1.5
**30°**	30′	0.51 ± 0.14 ^a1^	2.48 ± 0.11 ^b1^	4.12 ± 0.54 ^c1^
	60′	0.27 ± 0.02 ^a1^	3.52 ± 1.69 ^b1^	3.24 ± 1.15 ^b1^
	90′	0.25 ± 0.12 ^a1^	3.46 ± 0.15 ^b1^	2.26 ± 0.24 ^b1^
**40°**	30′	3.22 ± 0.78 ^a1^	3.24 ± 0.47 ^a1^	2.45 ± 0.02 ^a1^
	60′	3.26 ± 0.03 ^c1^	2.54 ± 0.00 ^b1^	2.44 ± 0.03 ^a1^
	90′	3.26 ± 0.35 ^a1^	2.57 ± 0.08 ^a1^	2.67 ± 0.41 ^a1^
**50°**	30′	2.00 ± 0.14 ^a1^	2.22 ± 0.25 ^b1^	3.28 ± 0.21 ^b1^
	60′	2.25 ± 0.02 ^a1^	3.30 ± 0.18 ^a1^	3.32 ± 0.62 ^a1^
	90′	2.78 ± 0.53 ^a1^	2.97 ± 0.08 ^a1^	2.59 ± 0.10 ^a1^

± Standard deviation is shown. *n* = 3. Different letters (^a,b,c^) in the same column indicate significant differences between times (*p* < 0.05). Different numbers (^1,2^) in the same row indicate significant differences between enzyme ratios (*p* < 0.05).

**Table 6:** Fatty acid profile of rainbow trout fillet, waste, and optimized fish protein hydrolyzate by-product fish oils.

	Fillet	Waste	Bph By-Product
**Ʃ** **SFA**	20.94 ± 0.67 ^ab^	18.81 ± 1.54 ^a^	22.15 ± 0.15 ^b^
**Ʃ** **MUFA**	31.48 ± 0.20 ^a^	40.72 ± 2.74 ^ab^	46.31 ± 0.17 ^b^
**Ʃ** **PUFA**	39.39 ± 0.73 ^b^	19.11 ± 1.65 ^a^	23.52 ± 0.03 ^ab^
**n6**	18.57 ± 1.12 ^a^	17.83 ± 1.29 ^a^	22.89 ± 0.03 ^b^
**n3**	19.71 ± 0.78 ^b^	9.92 ± 1.35 ^a^	6.99 ± 0.05 ^a^
**n6/n3**	0.95 ± 0.11 ^a^	1.80 ± 0.92 ^ab^	3.27 ± 1.17 ^b^

± Standard deviation is shown. *n* = 3. Different letters (^a,b^) in the same column indicate significant differences (*p* < 0.05).

## Data Availability

The data generated and analyzed during this study are included in this article.
